# Successful Lumpectomy in a Patient With Multicentric Breast Cancer

**DOI:** 10.7759/cureus.10072

**Published:** 2020-08-27

**Authors:** Quan D Nguyen, Anahita Tavana, Sarfaraz Sadruddin, Celia Chao

**Affiliations:** 1 Radiology, University of Texas Medical Branch, Galveston, USA; 2 Breast Surgery, University of Texas Medical Branch, Galveston, USA

**Keywords:** multicentric breast cancer, mastectomy, lumpectomy, breast conserving

## Abstract

When there is extensive breast cancer, patients typically undergo mastectomy. However, lumpectomy may still be performed for patients who are motivated to avoid a mastectomy and understand the risk for positive margins requiring second surgery in unique cases. This report details the surgical management and clinical reasoning behind lumpectomy for a multicentric breast cancer spanning 5 cm. The lumpectomy was a success with negative margins on final pathology.

## Introduction

Lumpectomy or mastectomy is the surgical management for breast cancer. The breast surgeon and the patient discuss the goals for each therapy and the risks and benefits associated with the decision. The arguments for lumpectomy are good cosmetic effects with minimal scar, avoidance of reconstructions and prosthesis, and minimal breast tissue excision. The arguments for mastectomy are lower recurrence rate, no need for radiation therapy, and no further surgery necessary [1].

The factors that favor lumpectomy include smaller, monocentric tumors; younger age; treatment carried out in specialized institutions; favorable physical factors; wire or radioactive seed localization of tumor; and patient compliance [1]. Typical contraindications for lumpectomy include locally widespread disease, multicentricity, diffuse (malignant) microcalcifications, first or second trimester of pregnancy, mutations in the *BRCA1* and *BRCA2 *genes, and an already irradiated thoracic wall [2].

## Case presentation

**Figure 1:**
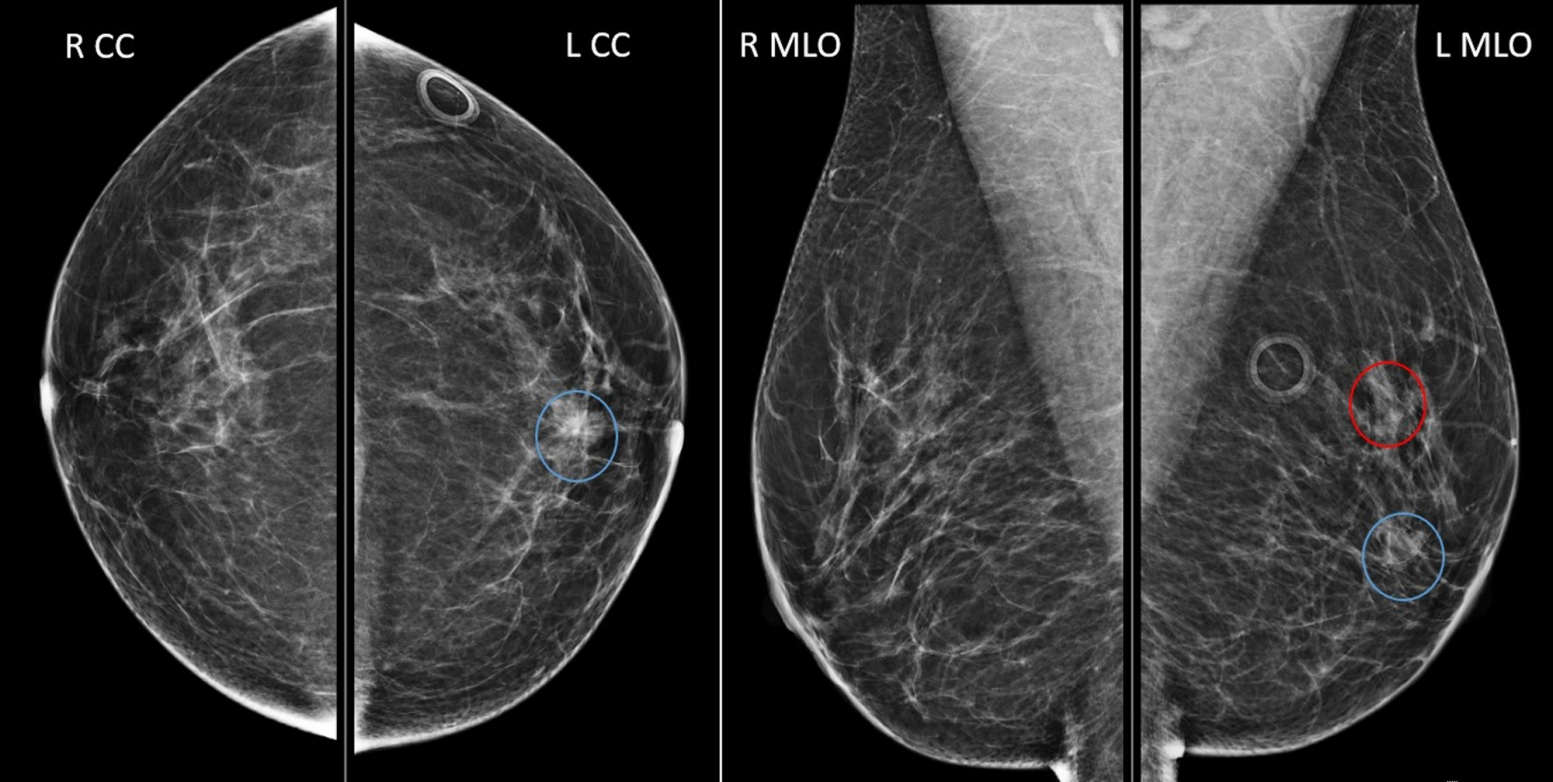
Screening mammogram 6 mm asymmetry in the upper region (red circle); 10 mm focal asymmetry in the central breast, 2 cm from nipple (blue circles); craniocaudal (left image) and mediolateral oblique (right image) views. R CC, right craniocaudal; L CC, left craniocaudal; R MLO, right mediolateral oblique; L MLO, left mediolateral oblique

The patient was a 55-year-old female who underwent screening mammogram and was found to have left breast 6 mm asymmetry in the upper region and a 10 mm focal asymmetry in the central breast (Figure 1). At the time of screening mammogram, the patient was asymptomatic with no palpable masses, changes in shape or volume of breast, or any nipple changes or discharge. Breast exam was unremarkable as well, with no dimpling, palpable masses, nipple retraction, or nipple discharge, and no cervical, supraclavicular, or axillary lymphadenopathy.

**Figure 2:**
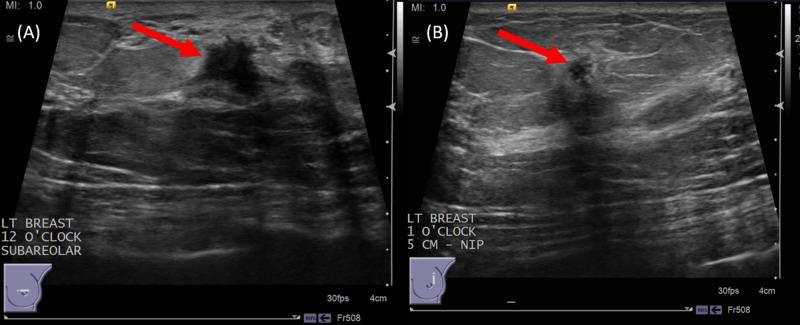
Left breast diagnostic ultrasound (A) Irregular mass measuring 10 x 6 x 8 mm on ultrasound at subareolar 12 o'clock region (red arrow); findings represent a suspicious abnormality, Breast Imaging-Reporting and Data System (BI-RADS) category 4C. (B) Irregular mass measuring 5 x 4 x 5 mm on ultrasound at 1 o'clock region, 5 cm from nipple (red arrow); findings represent a suspicious abnormality, BI-RADS category 4B.

**Figure 3:**
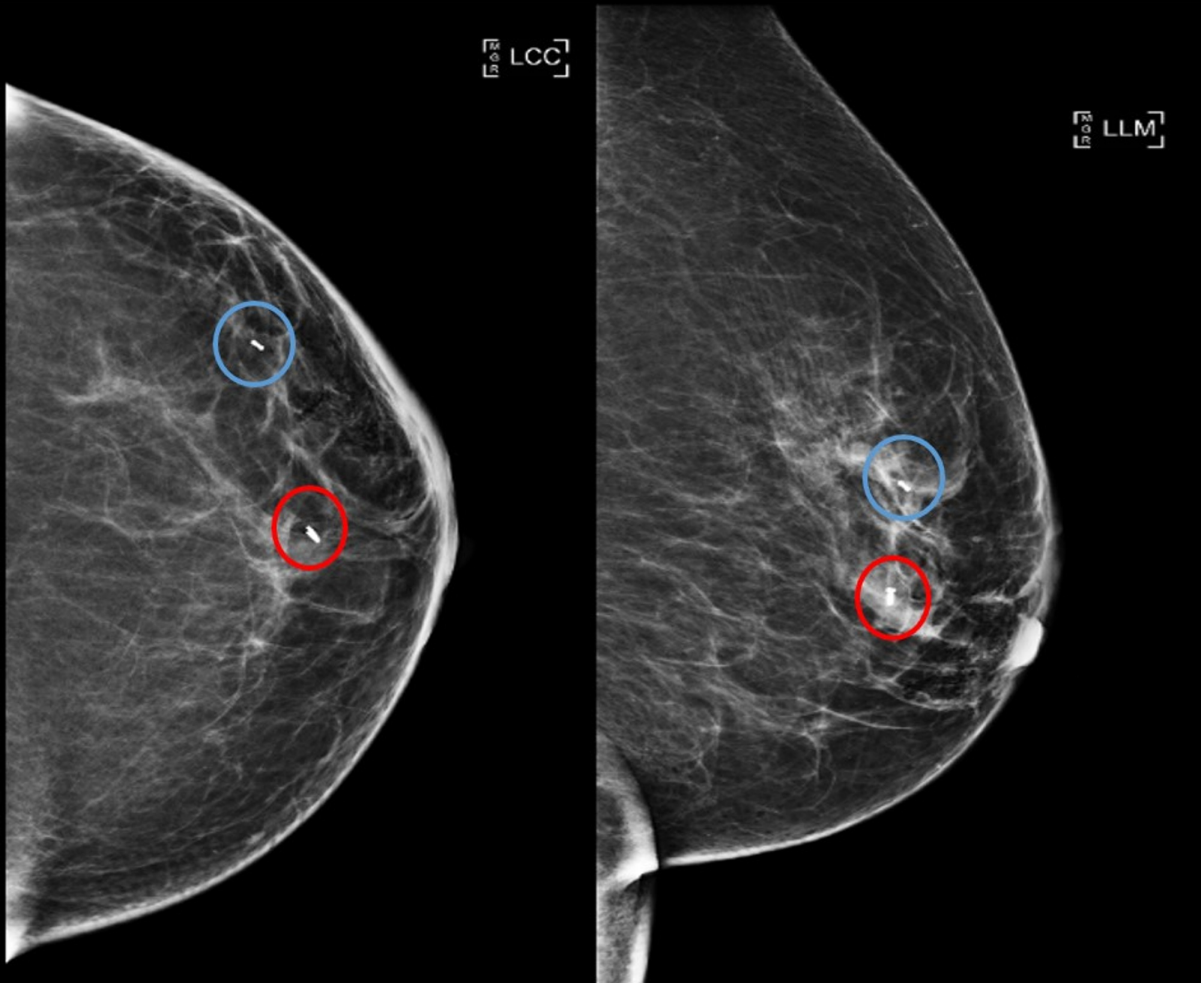
Left breast post-biopsy mammogram with biopsy marker Subareolar 12 o'clock region biopsied mass with ring-shaped biopsy marker (red circles). 1 o’clock 5 cm from nipple biopsied mass with barbell-shaped biopsy marker (blue circles). Craniocaudal (left image) and lateromedial (right image) views. LCC, left craniocaudal; LLM, left lateromedial

Further workup with diagnostic ultrasound of left breast demonstrated an irregular mass measuring 10 mm at subareolar 12 o’clock region and an irregular mass measuring 5 mm at 1 o’clock region, 5 cm from nipple (Figure 2). Ultrasound-guided core needle biopsies were performed, and biopsy markers were placed. Post-biopsy mammogram demonstrated the two masses spanning 5 cm (Figure 3).

**Figure 4:**
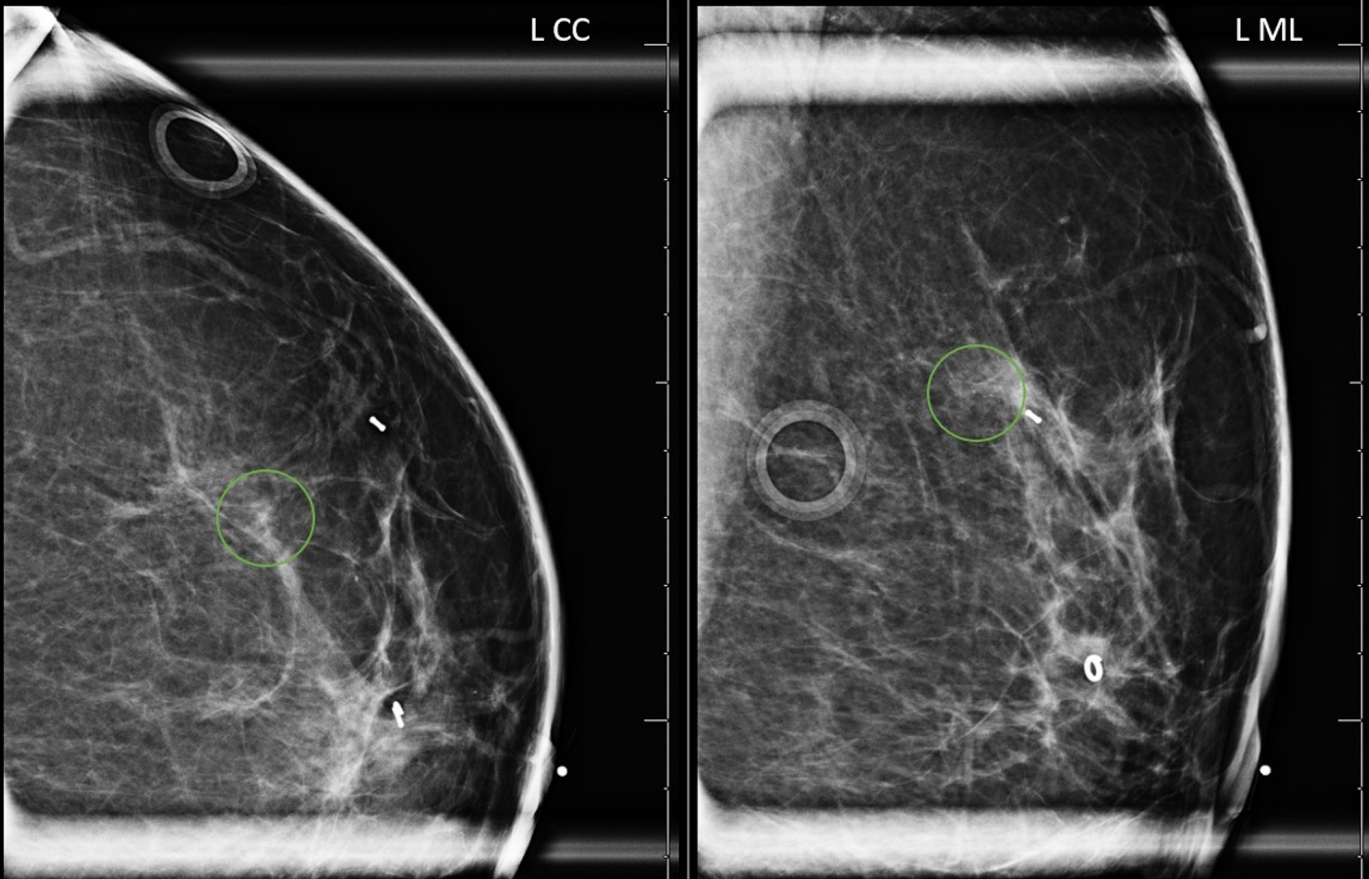
Left breast diagnostic mammogram for workup of calcifications Grouped amorphous calcifications measuring 5 mm at 1 o’clock region, 6 cm from nipple (green circles). Craniocaudal (left image) and mediolateral (right image) views. L CC, left craniocaudal; L ML, left mediolateral

**Figure 5:**
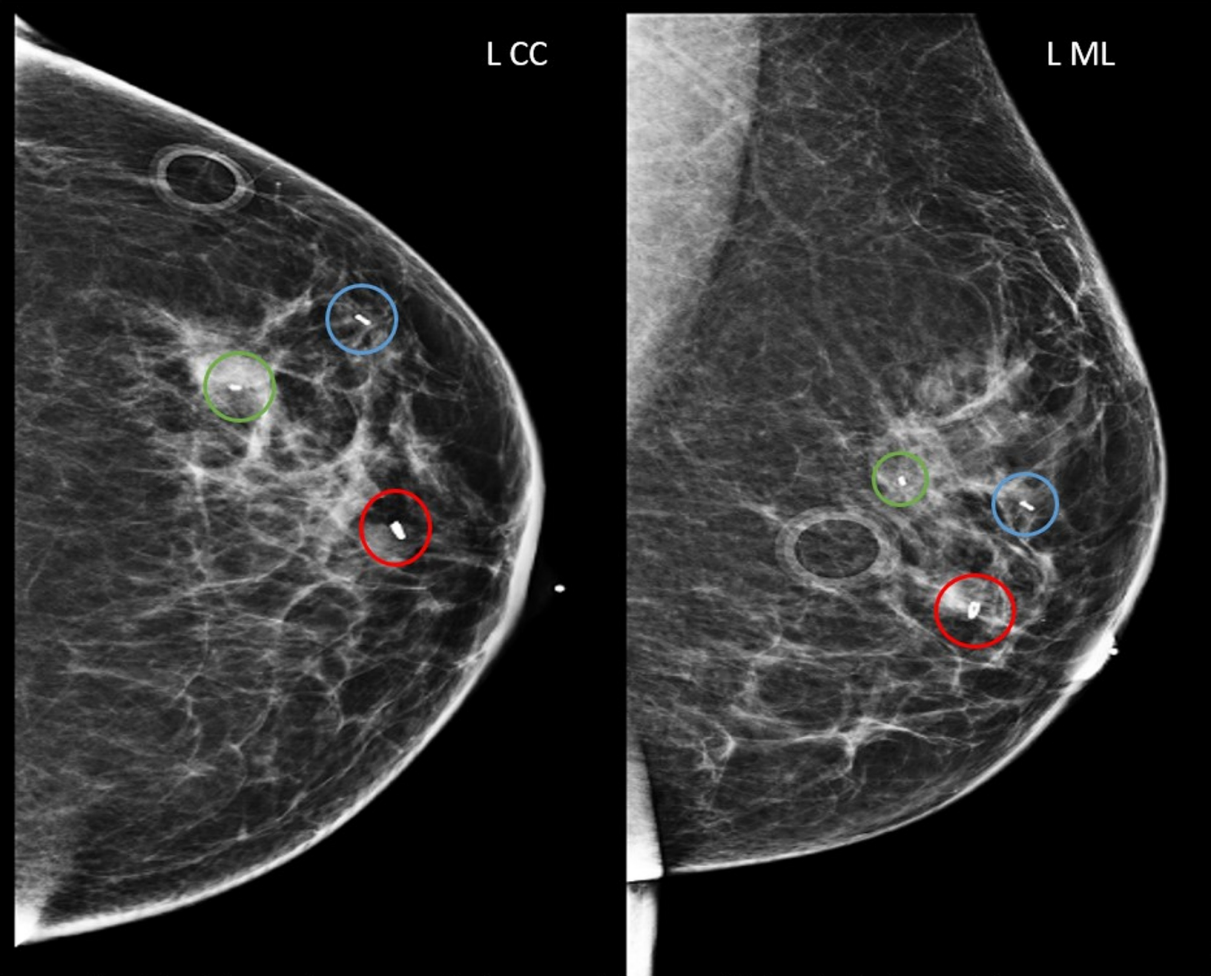
Left breast post-biopsy mammogram with biopsy markers Subareolar 12 o'clock region biopsied mass with ring-shaped biopsy marker (red circles). 1 o’clock 5 cm from nipple biopsied mass with barbell-shaped biopsy marker (blue circles). 1 o’clock 6 cm from nipple biopsied calcifications with bar-shaped biopsy marker (green circles). Craniocaudal (left image) and mediolateral (right image) views. L CC, left craniocaudal; L ML, left mediolateral

Pathology revealed invasive ductal carcinoma (IDC) for both the left breast subareolar 12 o’clock mass and the 1 o’clock mass. In addition, diagnostic mammogram demonstrated grouped calcifications measuring 5 mm at 1 o’clock region, 6 cm from nipple with stereotactic-guided core biopsy revealing high-grade ductal carcinoma in situ (DCIS) (Figure 4). The tumors were, by imaging and biopsies, clinical stage IA. Post-biopsy mammogram demonstrated the two masses and additional calcifications still spanning 5 cm in greatest dimension (Figure 5).

**Figure 6:**
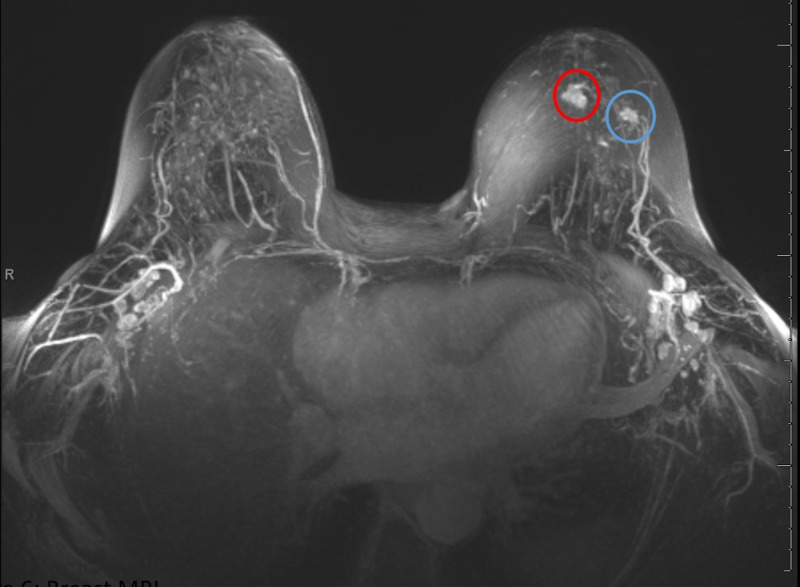
Breast MRI Left breast enhancing irregular masses corresponding with the mammographic sites of biopsy-proven invasive ductal carcinoma (IDC), measuring 15 mm at 12 o'clock subareolar region (red circle) and measuring 10 mm at 1 o'clock region, 5 cm from nipple (blue circle). There is no abnormal enhancement at the site of biopsy-proven ductal carcinoma in situ (DCIS) in the upper outer quadrant, 5 cm from nipple.

MRI of bilateral breasts was obtained to evaluate the extent of the disease and to help the patient and the surgeons determine whether it would be more appropriate to proceed with lumpectomy or mastectomy (Figure 6). No further site of disease was identified.

The patient’s options for surgery were either mastectomy with a sentinel lymph node biopsy (SLNB) or lumpectomy with oncoplastic reconstruction and an SLNB. The patient understood the implications of having a lumpectomy versus mastectomy, including possible need for further surgery with a re-lumpectomy or a completion mastectomy, as well as the need for adjuvant radiation therapy, hormonal therapy for 5-10 years, and adjuvant biological therapy or chemotherapy based on final pathology.

**Figure 7:**
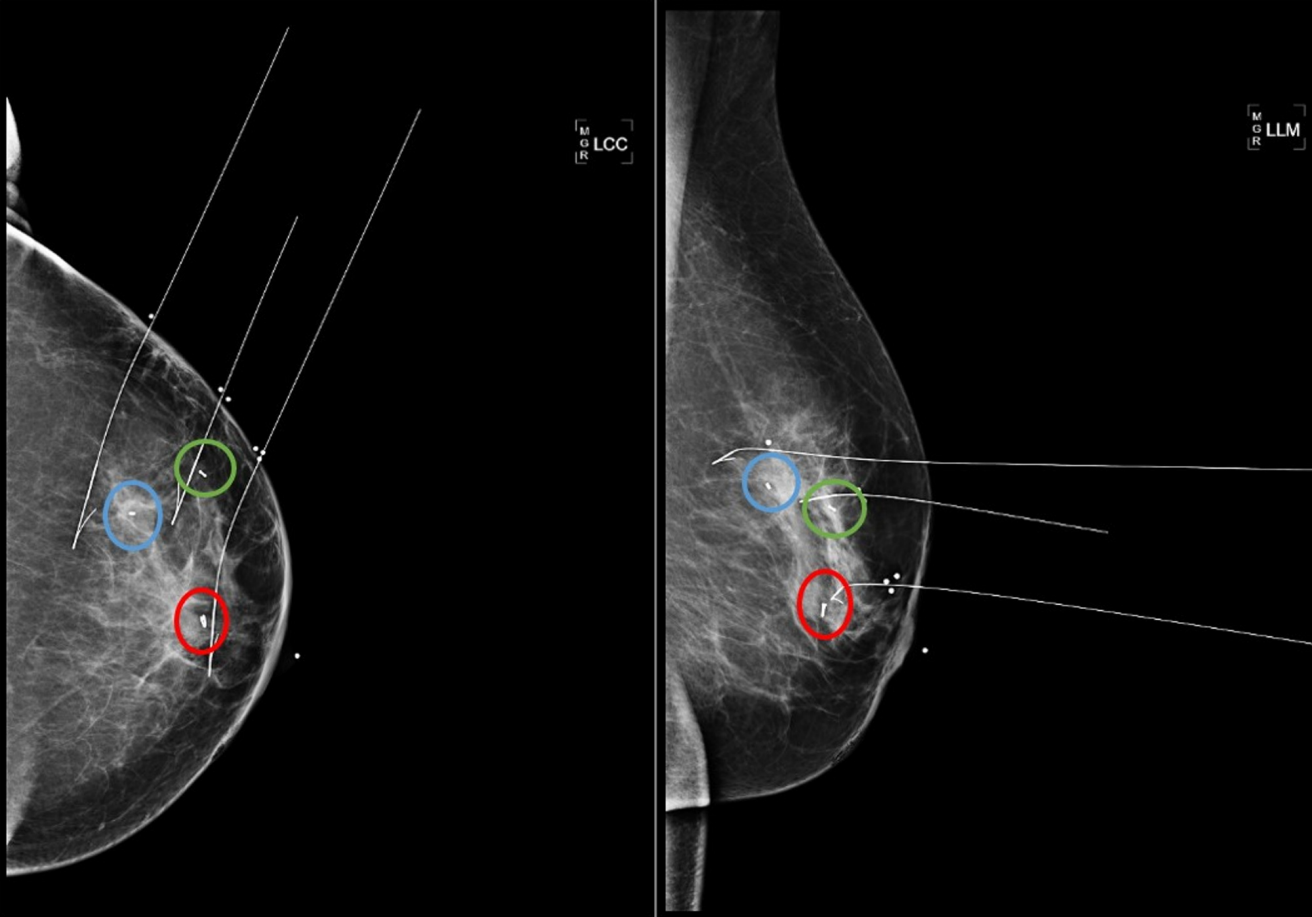
Left breast mammogram with wire localization for surgical lumpectomy Subareolar 12 o'clock region biopsied mass with ring-shaped biopsy marker (red circles). 1 o’clock 5 cm from nipple biopsied mass with barbell-shaped biopsy marker (blue circles). 1 o’clock 6 cm from nipple biopsied calcifications with bar-shaped biopsy marker (green circles). Craniocaudal (left image) and lateromedial (right image) views. LCC, left craniocaudal; LLM, left lateromedial

**Figure 8:**
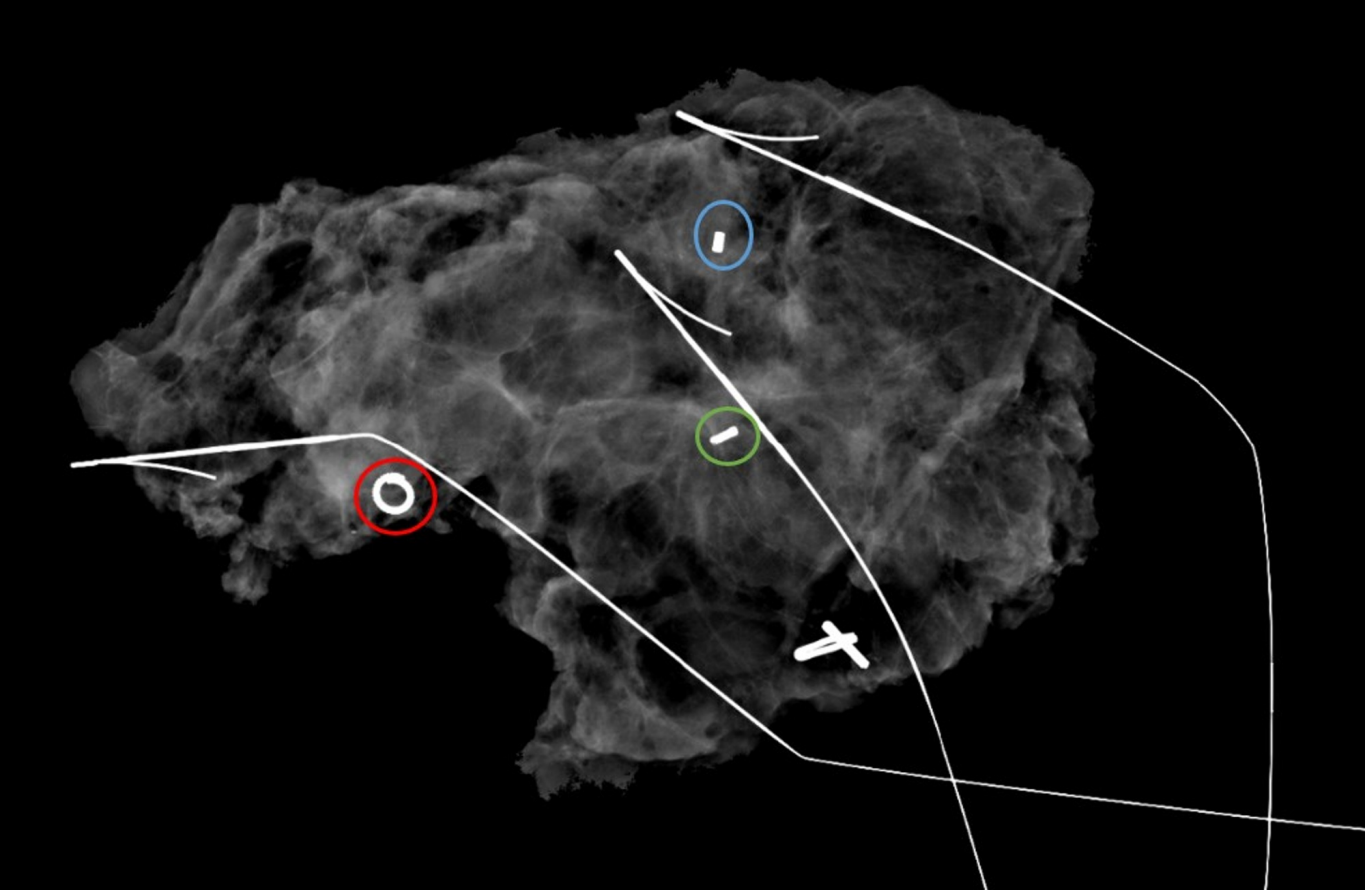
Lumpectomy specimen radiograph Subareolar 12 o'clock region biopsied mass with ring-shaped biopsy marker (red circle). 1 o’clock 5 cm from nipple biopsied mass with barbell-shaped biopsy marker (blue circle). 1 o’clock 6 cm from nipple biopsied calcifications with bar-shaped biopsy marker (green circle).

The patient's ultimate decision was to undergo breast-conserving surgery with a left wire-guided partial mastectomy, an SLNB, and oncoplastic reconstruction. Three-wire mammogram-guided localization was performed targeting the three biopsy markers (Figure 7). Lumpectomy successfully removed the three sites of malignancy with surgical specimen containing all the biopsy markers (Figure 8). Surgical pathology report confirmed negative surgical margins; SLNB detected metastases in three sentinel lymph nodes. Pathology from the 1 o’clock lesion revealed intermediate-grade DCIS at the site of calcifications, along with IDC, grade 2, and human epidermal growth factor receptor 2 (HER2) negative (1+); pathology from the subareolar 12 o’clock lesion revealed intermediate-grade DCIS, as well as IDC, grade 2, estrogen receptor (ER) 50, progesterone receptor (PR) 0, and HER2 equivocal (2+) with no amplification on fluorescence in situ hybridization (FISH). CT of chest, abdomen, and pelvis showed no evidence of malignancy.

## Discussion

Our case presented a 55-year-old female who underwent successful lumpectomy despite having multicentric breast cancer spanning 5 cm in greatest dimension. The case demonstrated that a distance of 5 cm between two clips does not warrant mastectomy in every case. If there are two lesions very far apart in different quadrants, separate lumpectomies could be performed with clear margins, followed by radiation [3]. Patients should be made aware of an approximately 30% chance of positive margins requiring margin re-excision; however, the chance of conversion to mastectomy due to positive margins is only around 7% [4]. This particular patient was highly motivated to avoid mastectomy. Furthermore, the institution had plastic and breast surgeons who had experience in performing oncoplastic surgery with reasonable success, allowing for a team approach with both surgeons performing their mutual skills for the patient. Following lumpectomy, patients will have tissue rearrangement and a smaller cup size; however, they generally prefer that to wearing a breast prosthesis on an insensate chest wall or reconstruction with no sensation on the breast envelope. In addition, the patient had an area in her cancer that was HER2 positive; if oncology considers upfront chemotherapy, that is further rationale to consider lumpectomy if she responds. Following the initial management of primary breast cancer, current clinical guidelines recommend annual follow-up with screening mammography. Screening ultrasound is not recommended for breast cancer surveillance by most guidelines, and further studies are required to determine which subgroup of patients would benefit most from surveillance with MRI [5]. Therefore, our patient was advised to undergo annual screening mammogram to monitor for recurrence of her breast cancer.

Surgical options for multicentric breast cancer have evolved in recent years. Historically, primary tumors in different quadrants of the same breast or tumors greater than 5 cm in size would be managed by total mastectomy. In recent years, however, there has been an encouraging upward trend in the use of breast-conserving surgeries (i.e. lumpectomies); more women are being offered surgical options that were once considered to be contraindicated but are now found to provide equivalent breast cancer-specific survival rates as mastectomy [6].

In addition, the molecular phenotype with appropriate chemotherapy has become more important for patient survival than local control with surgery in recent years. Moreover, patients now receive radiation for much less time and toxicity for equal benefit in comparison to the previous protocol of six weeks of radiation. Therefore, recent advances in chemotherapy and the shortened courses of radiotherapy could motivate both surgeons and patients to consider lumpectomy for the management of multicentric breast cancers rather than mastectomy.

## Conclusions

This case demonstrates a patient making an informed decision with her breast surgeon to undergo a lumpectomy rather than mastectomy in a case of extensive breast cancer spanning 5 cm. Breast-conserving surgery is still an option for extensive breast cancer when the patient is highly motivated to avoid mastectomy, the breast surgeon feels confident that there is a chance for negative surgical margins, and the plastic surgeon is able to perform oncoplastic surgery with reasonable success.
